# 415. A Whole Genome Sequencing Analysis of a Multi-unit Long-term Care Facility COVID-19 Outbreak

**DOI:** 10.1093/ofid/ofab466.615

**Published:** 2021-12-04

**Authors:** Ling Yuan Kong, Leighanne Parkes, Yves Longtin, Christina Greenaway, Jerry Zaharatos, Vivian Loo, Noémie Savard, Réjean Dion, Fournier Éric, Michel Roger, Sandrine Moreira

**Affiliations:** 1 SMBD Jewish General Hospital, Montreal, Quebec, Canada; 2 Jewish General Hospital, Montreal, Montreal, QC, Canada; 3 Jewish General Hospital, McGill University, Montreal, Montreal, QC, Canada; 4 McGill University, Montreal, Quebec, Canada; 5 Direction de la santé publique de Montréal, Montreal, Quebec, Canada; 6 Institut national de la santé publique du Québec, Sainte-Anne-de-Bellevue, Quebec, Canada

## Abstract

**Background:**

The coronavirus disease (COVID-19) pandemic has affected residents in long-term care facilities (LTCF) significantly. Understanding transmission dynamics in this setting is crucial to control the spread of COVID-19 in this population. Using whole genome sequencing (WGS) of SARS-CoV-2, we aimed to delineate the points of introduction and transmission pathways in a large LTCF in Quebec, Canada.

**Methods:**

Between 2020-10-28 and 2021-01-09, COVID-19 cases occurred in 102 residents and 111 HCW at a 387-bed LTCF; cases were distributed in 11 units on 6 floors. As part of outbreak analysis, SARS-CoV-2 isolates underwent WGS using the Oxford Nanopore Minion and the Artic V3 protocol. Lineage attribution and sequence types (ST, within 3 mutations) were assigned based on Pangolin classification and variant analysis. Epidemiologic data including date of positive PCR test, resident room number and HCW work location were collected. Self-reported high-risk exposures were collected by HCW questionnaire via phone interview after consent. Cases and their ST, geo-temporal relations and HCW-reported exposures were examined via network plots and geography-based epidemic curves to infer points of introduction and paths of transmission.

**Results:**

Of 170 isolates available from 100/102 residents and 70/111 HCW, 130 (76.4%) were successfully sequenced. Phylogenetic analysis revealed 7 separate introductions to the LTCF. Grouping of ST by units was observed, with temporal appearance of ST supporting HCW introduction in 7/11 units. Proportion of phone interview completion was low at 35% (26/70). Few HCW recalled specific high-risk exposures. Recalled exposures supported by genetic linkage revealed potential between-unit introductions from HCW-to-HCW transmission at work and outside the workplace (e.g. carpooling). On one unit, a wandering resident was identified as a likely source of transmission to other residents (Figure 1).

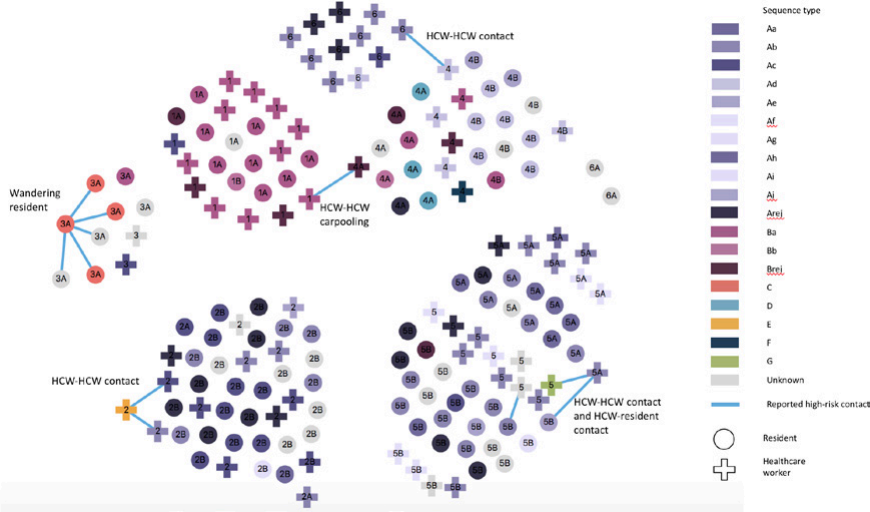

Network plot of cases clustered by geographic unit, colour-coded by sequence type. Circles represent residents; addition signs represent healthcare workers. Blue lines represent identified high-risk exposures. Node labels represent floor and unit identifiers; 2 units per floor.

**Conclusion:**

We demonstrate the complex genomic epidemiology of a multi-unit LTCF outbreak, putting into evidence the importance of a multi-faceted approach to limit transmission. This analysis highlights the utility of using WGS to uncover unsuspected transmission routes, such as HCW contact outside work, which can prompt new infection control measures.

**Disclosures:**

**All Authors**: No reported disclosures

